# Expression profile of endothelin receptors (ET_A_ and ET_B_) and microRNAs-155 and -199 in the corpus cavernosum of rats submitted to chronic alcoholism and diabetes mellitus

**DOI:** 10.1590/1414-431X20176329

**Published:** 2018-03-01

**Authors:** F.Z. Gonçalves, F.S. Lizarte, P.C. Novais, D. Gattas, L.G. Lourenço, C.A.M. de Carvalho, D.P.C. Tirapelli, C.A.F. Molina, L.F. Tirapelli, S. Tucci

**Affiliations:** 1 Departamento de Cirurgia e Anatomia, Faculdade de Medicina de Ribeirão Preto, Universidade de São Paulo, Ribeirão Preto, SP, Brasil; 2 Departamento de Ciências da Saúde, Universidade de Marília, Marília, SP, Brasil; 3 Instituto de Ciências Biológicas e da Saúde, Universidade Federal de Alagoas, Maceio, AL, Brasil

**Keywords:** Alcoholism, Corpus cavernosum, miRNAs, Endothelin receptors

## Abstract

Recent evidence shows that chronic ethanol consumption increases endothelin (ET)-1 induced sustained contraction of trabecular smooth muscle cells of the corpora cavernosa in corpus cavernosum of rats by a mechanism that involves increased expression of ET_A_ and ET_B_ receptors. Our goal was to evaluate the effects of alcohol and diabetes and their relationship to miRNA-155, miRNA-199 and endothelin receptors in the corpus cavernosum and blood of rats submitted to the experimental model of diabetes mellitus and chronic alcoholism. Forty-eight male Wistar rats were divided into four groups: control (C), alcoholic (A), diabetic (D), and alcoholic-diabetic (AD). Samples of the corpus cavernosum were prepared to study the protein expression of endothelin receptors by immunohistochemistry and expression of miRNAs-155 and -199 in serum and the cavernous tissue. Immunostaining for endothelin receptors was markedly higher in the A, D, and AD groups than in the C group. Moreover, a significant hypoexpression of the miRNA-199 in the corpus cavernosum tissue from the AD group was observed, compared to the C group. When analyzing the microRNA profile in blood, a significant hypoexpression of miRNA-155 in the AD group was observed compared to the C group. The miRNA-199 analysis demonstrated significant hypoexpression in D and AD groups compared to the C group. Our findings in corpus cavernosum showed downregulated miRNA-155 and miRNA-199 levels associated with upregulated protein expression and unaltered mRNA expression of ET receptors suggesting decreased ET receptor turnover, which can contribute to erectile dysfunction in diabetic rats exposed to high alcohol levels.

## Introduction

Erectile dysfunction (ED) is defined by the National Institutes of Health and American Urological Association as the persistent inability to achieve or maintain an erection suitable for sexual satisfaction (1). Projections for 2025 expect a prevalence of 322 million men with some type of erectile dysfunction (2).

The main risk factors for ED include advanced age, hypertension, overweight, obesity, sedentary lifestyle, metabolic syndrome, diabetes, and atherosclerosis (3). It is believed that there may be a relationship between chronic alcoholism and ED (4).

The mechanisms related to erection are complex and interconnected. In the absence of sexual stimulation, the penis is flaccid. A contraction effect of the vasculature and the cavernous tissue occurs mainly in response to noradrenaline and endothelin-1 (ET-1), which is a potent vasoconstrictor peptide acting on its receptors (ET_A_ and ET_B_) and might be related to the pathophysiology of ED. *In vivo* and *in vitro* studies have demonstrated contraction of the corpus cavernosum mediated by ET_A_ while ET_B_ triggered vasodilation (5,6).

Published studies demonstrate an increase in ET-1 in the blood of impotent men, as well as in diabetic patients and in rats submitted to chronic alcohol consumption (7–9). In addition to vasoconstriction, ET-1 also has mitogenic and inflammatory properties and its increase is related to numerous pathological processes such as cardiovascular diseases, arterial hypertension and chronic renal failure (10).

MicroRNAs are defined as non-coding RNAs consisting of 19 to 25 nucleotides, cleaved in the cytoplasm from precursors with 60 to 110 nucleotides (pre-miRs) by the enzyme RNA polymerase III, called Dicer (11–13). miRNAs regulate gene expression by reducing the levels of mRNA transcripts, translated proteins, or both. Many miRNAs are conserved between remotely related organisms, suggesting that these molecules are essential in the regulation of several cellular processes such as differentiation, proliferation, apoptosis and metabolism. Consequently, loss of the regulation from miRNA expression may result in a number of disorders (14).

The objective of our study was to evaluate the effects of alcohol, diabetes and their association on miRNA-155 and miRNA-199 and endothelin receptors (ET_A_ and ET_B_) in the corpus cavernosum and blood of rats submitted to the experimental model of diabetes mellitus and "semi-voluntary chronic alcoholism", and investigate whether there is a relationship between miRNAs and endothelin receptors.

## Material and Methods

A total of 48 male Wistar rats (*Rattus norvegicus*) were obtained from the Faculty of Medicine of Ribeirão Preto, Universidade de São Paulo, after approval by the Ethics Committee of the institution. Animals were divided in 4 groups and followed by 4 weeks after the adaptive period: control group (C), alcoholic group (A), diabetic group (D), alcoholic-diabetic group (AD), all groups with 12 animals each. Previous studies with similar design showed that the number of animals is considered adequate to evaluate the expression of genes and miRNAS in the corpus cavernosum of rats submitted to chronic alcoholism and diabetes mellitus (15–17).

For the animals in the alcohol groups (A, AD), the model of "semi-voluntary alcoholism" was used, in which 20% ethanol solution was the only liquid available to these animals, following a model proposed by Tirapelli et al. (18). Briefly, the animals went through an adaptive phase consisting of supplying ethanol in increasing weekly concentrations of 5, 10, 20%, starting the experimental phase after the third week of treatment. At the end of the third week, diabetes was induced in rats from the D and AD groups by one *iv* injection of alloxan (dissolved in 0.1 citrate buffer, 45 mg/kg) into the tail vein. The control group received citrate buffer only. Weekly blood samples were taken from a tail stab to measure the level of blood glucose using reagent strips (BM-Accutest® and an auto-recorder, Accutrend®, Boehring Mannheim, UK). Rats in the control, alcoholic, diabetic and alcoholic-diabetic groups were weighed at induction of diabetes and at the end of the experimental period. The rats of all groups were followed for 7 weeks and then euthanized.

For immunohistochemistry, the corpus cavernosum of 6 animals from the control, alcoholic, diabetic, and alcoholic-diabetic groups were immediately removed and fixed for 24h in ice-cold 0.1 mol/L PBS, pH 7.4, containing 4% paraformaldehyde, followed by cryoprotection in 15% of sucrose for 4h and 30% sucrose overnight at 4°C. Longitudinal sections (10 µm) of the corpus cavernosum were immunohistochemically analyzed via avidin-biotin-peroxidase (Novostain Super ABC Kit, Universal, NCL-ABCu, Novocastra Laboratories Ltd., UK) - (Universal Kit Mach 4 Biocare Medical, USA). The sections were incubated with 3% H_2_O_2_, followed by antigen retrieval with a moist steam cooker Optistream Plus (Krups North America, Inc., USA) with 10 mM citrate buffer at pH 6.0 for 35 min. Then, the sections were incubated for 24 h with primary antibodies for ET_A_ and ET_B_ diluted 1/300 in PBS solution of bovine serum albumin. Subsequently, the blocking of the endogenous biotin was performed (Biotin Blocking System, Dako North America, Inc., USA) and only then the sections were incubated with secondary antibody HRP kit MACH 4-Universal Polymer (M4BD534, Biocare Medical) and then with avidin-biotin-peroxidase kit (1/200 in PBS). Color was developed by the addition of diaminobenzidine (Sigma, USA).

The sections were dehydrated in ethanol, cleared with xylene and mounted under a cover slip with Permount liquid (Fisher Scientific Company LLC, USA).

To evaluate the background reaction, the procedures were also performed in sections incubated only with the secondary antibodies (indirect technique) or in the absence of antibodies (direct technique). The slides for immunohistochemistry were analyzed using the Zeiss microscope Axioskop 2 plus model at ×400 magnification. The number of cells with positive staining for ET_A_ and ET_B_ receptors was measured by using a camera (Axio Cam, Zeiss, Germany) and the program Axiovision 4.6 (Zeiss).

### Expression profile of miRNAs-155 and -199

The expression profile of the miRNAs-155 and -199 were analyzed in blood and cavernous tissue samples from each animal. Total cellular RNA was extracted using Trizol Reagent (Invitrogen, USA) and RNA was reverse transcribed to single-stranded cDNA, using a High Capacity Kit (Applied Biosystems, USA) according to the manufacturer's protocol. For quantitative analysis of the miRNAs-155 and -199, we used the commercially available system TaqMan Assay-ondemand (Applied Biosystems). Reverse transcription was performed using 5 ng total RNA for each sample in 7.5 µL of the total reaction mixture. The cDNA obtained was diluted 1:4 and 4.5 µL was used for each 10 µL of the quantitative real-time polymerase chain reaction mixture using the TaqMan Master Mix (Applied Biosystems). All reactions were carried out in duplicate and analyzed with the 7500 Sequence Detection System apparatus (Applied Biosystems). Data were analyzed using the ABI-7500 SDS software. The total RNA absorbed was normalized based on the Ct value for U6 (000391). The variation in expression among samples was calculated by the 2-ΔΔCt method, with the mean ΔCt value for a group of 6 samples from control rats used as a calibrator.

### Statistical analysis

For the evaluation of all studies in this research (protein expression and gene expression), statistical analysis was performed using the Kruskal-Wallis test and Dunn's multiple comparison post-test. We used the GraphPad Prism program 6:00 version for Windows (GraphPad Software, USA) and considered statistically significant if P<0.05.

## Results

After induction of diabetes, the diabetic and alcoholic-diabetic rats had similar weights of 322±15 and 359±13 g, respectively (P>0.05), but they weighed significantly less (P<0.005) than the control and alcoholic rats (515±19 and 430±21 g). Serum glucose concentrations were significantly higher (P<0.01) in the diabetic (456±23 mg/dL) and ethanol-diabetic groups (440±16 mg/dL) than in control and alcoholic groups (102±8 and 108±5 mg/dL, respectively).

### Immunohistochemistry

**Figure 1.**
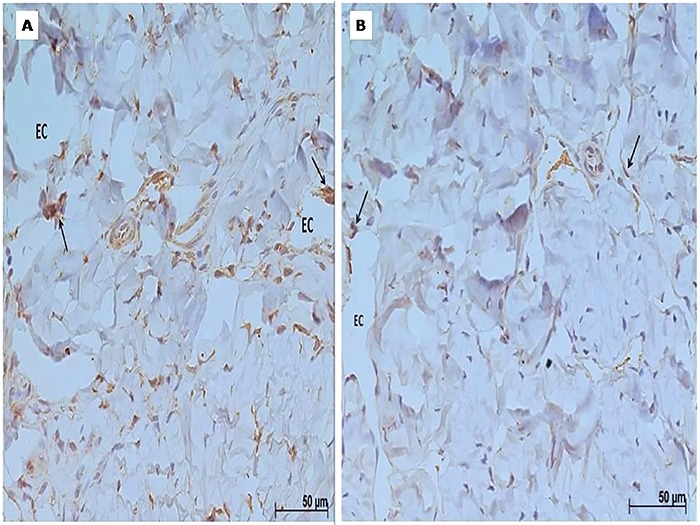
Representative immunohistochemical photomicrography of the overall expression of endothelin B (*Panel A*) and A (*Panel B*) protein in cross-section of the rat cavernous bodies of the alcoholic-diabetic group. Cavernous spaces (EC) and positive markings (arrows) are indicated. Magnification: 400×. The expression of endothelin B and A receptors in corpus cavernosum muscle can be observed in brown.

The rat cavernosal smooth muscle (CSM) had intense staining for ET_A_ and ET_B_ receptors. Immunostaining for ET_A_ and ET_B_ was markedly higher in the ethanol, diabetic and alcoholic-diabetic groups than in the control group (Figure 1).

**Figure 2.**
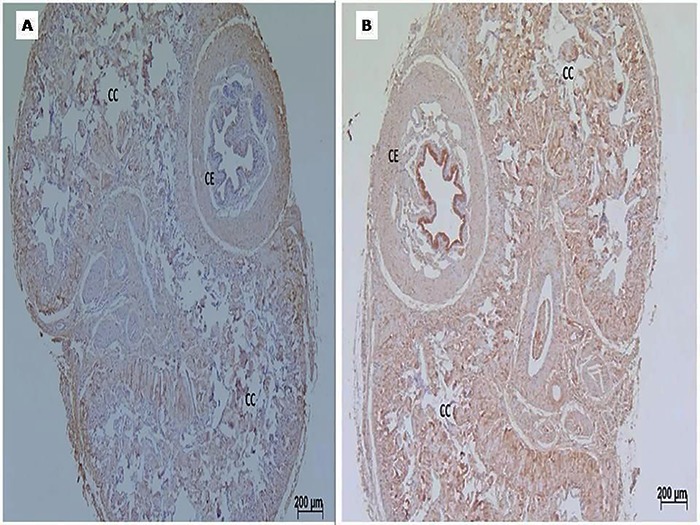
Representative immunohistochemical photomicrography of the overall expression of endothelin B (*Panel A*) and A (*Panel B*) protein in cross-section of the rat cavernous bodies of the alcoholic-diabetic group. Diffuse marking on the erectile tissue can be seen, especially close to the tunica albuginea for endothelin B. Cavernous body (CC); Spongy body (CE). Magnification: 50×.

The distribution of ET_A_ and ET_B_ receptors was not homogeneous or proportional. ET_B_ receptors were concentrated in the periphery of the corpora cavernosa and in their transition with the tunica albuginea, whereas ET_A_ receptors were diffusely distributed and in a higher concentration throughout the corpus cavernosum (Figure 2).

### Expression profile of genes and miRNAs

**Figure 3.**
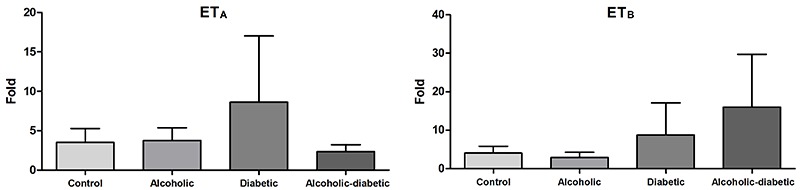
Mean (±standard error) of endothelin A (ET_A_) and B (ET_B_) expression in cavernous tissue samples of the groups studied. There were no significant differences among the groups (Kruskal-Wallis test).

We found no significant difference between the groups analyzed. There was a non-significant increase in the expression profile of the ET_A_ receptor in group D and the ET_B_ receptor in group AD both in relation to group C (Figure 3).

**Figure 4.**
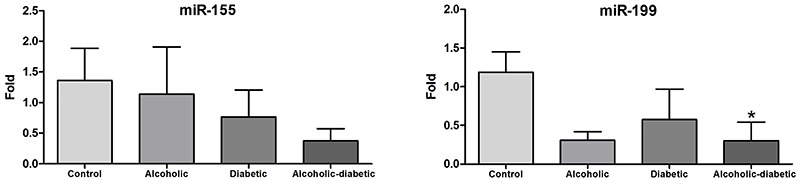
Mean (±standard error) of microRNAs-155 and -199 expression in the cavernous tissue samples of the groups studied. *P<0.05, compared to the control group (Kruskal-Wallis test, Dunn's post-test).

In the tissue of the corpus cavernosum, a significant downregulation of the miRNA-199 in the AD group and non-significant downregulation in the A and D groups compared to the C group were observed, whereas the miR-155 analysis demonstrated non-significant gene downregulation in the experimental groups compared to group C (Figure 4).

**Figure 5.**
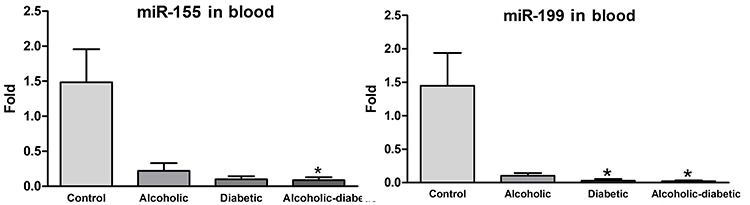
Mean (±standard error) of microRNA-155 and -199 expression in blood samples of the groups studied. *P<0.05, compared to the control group (Kruskal-Wallis test, Dunn's post-test).

When analyzing the blood microRNA profile, a significant downregulation of miR-155 was found in group AD and non-significant downregulation was observed in the other experimental groups compared to the C group. The miR-199 analysis demonstrated significant downregulation in groups D and AD compared to group C, in addition to non-significant downregulation in group A (Figure 5).

## Discussion

We used the experimental model for alcoholism proposed by Tirapeli et al. (18) and adopted the total time of seven weeks of follow-up since there are studies in the literature that used the same period (6,16,19). Another relevant aspect is that a longer follow-up time would lead to significant animal death due to the deleterious effects of alcoholism and diabetes.

The use of rats as an experimental model of ED and for molecular biology assays has been highlighted in the literature in recent decades. Current studies evidence ET receptor pathways as important and promising targets for new studies related to ED, alcoholism and diabetes (7–9). Lizarte et al. (16) showed that ethanol consumption significantly reduced testosterone levels, which is associated with sexual dysfunction and altered reactivity of cavernous smooth muscle in rats. Recent findings showed also that chronic ethanol consumption increases the expression of ET-1 and the contractile action of this peptide in the corpus cavernosum of rats. ET is a 21 amino-acid peptide that belongs to a potent vasoconstrictor family acting on its receptors (ET_A_ and ET_B_) both of which have been shown in previous animal and human studies with cavernous tissue. ET-1 induces a strong and sustained contraction of trabecular smooth muscles cells of the corpora cavernosa contributing to the maintenance of corpus cavernosum tone. Increased levels of ET-1 have been associated with ED in numerous pathological processes, especially in vascular disease related to hypercholesterolemia and diabetes mellitus (15,17). ET_A_ receptors are expressed in vascular smooth muscle cells and are related to vasoconstriction (20). However, the activation of endothelial ET_B_ receptors promotes vasodilation through the formation of NO and release of prostaglandin (21,22). Despite the high prevalence of alcoholism and diabetes, there are few studies related to alterations in endothelin receptors in penis erectile tissue.

Immunohistochemical analysis performed by Sullivan et al. (23) with alloxan-induced diabetic rabbits resulted in a significant increase in ET_B_ after 6 months of diabetes and a non-significant increase of ET_A_ diffused by the corpora cavernosa in the microvasculature located at the edge of the cavernosum corpus and tunica albuginea. Bell et al. (24) studied the effect of streptozotocin-induced diabetes in the cavernosum corpus of rats, through high-resolution autoradiography, and demonstrated an increase in ET and ET-1 after 2 months of diabetes onset.

Well-established studies have demonstrated an increase in the concentration of ET-1 in the blood of impotent men, as well as in diabetic patients and in rats submitted to chronic alcohol consumption, thus demonstrating the involvement of the receptor pathway of endothelin with those diseases (7–9).

Leite et al. (6) performed an experimental study using rats submitted to alcoholism with a similar study design to our research. The authors used real-time PCR and did not find an increase in gene expression of ET_A_ or ET_B_, similarly to our results. We also did not find alterations in the gene expression in groups D and AD by this technique. Also in the study by Leite et al. (6), the protein expression was analyzed by western blotting, demonstrating an increase of ET_A_ only in the alcoholized group compared to the control group. Similar findings in our studies show a greater increase of ET with respect to ET_B_ by immunohistochemical analysis, which would be relevant for the pathophysiology of ED associated with alcoholism, since ET_A_ has a more potent vasoconstricting action.

Other known ED factors, such as obesity and arterial hypertension, also have well-established models, such as a study by Carneiro et al. (25). In that study, rats submitted to mineralocorticoid-induced hypertension demonstrated that the contractile response to ET-1 was abolished by an ET_A_ receptor antagonist. The administration of an ET_B_ antagonist did not produce any significant effect on the vasoconstricting action of ET-1, while the use of ET_B_ agonist caused relaxation and not vasoconstriction in microtiter of cavernous bodies. Analysis by western blotting found a non-significant increase of ET_A_ and a significant decrease of ET_B_ and eNOS (25). Therefore, we can suggest that the pathophysiology of ED associated to hypertension has ET_A_ receptors expression profile similar to alcoholism, since in both ED risk factors there was an increase in ET_A_ receptors, which have a greater vasoconstricting effect.

Although not significant, in our study, the ET_B_ receptor presented higher expression in real-time PCR analysis. We can suggest a possible regulation of ET_B_ by miR-199 mainly in group AD, since miR-199 presented a significant difference in this group and an inverse expression pattern of ET_B_.

Studies have found a 2% change (increase or decrease) in miRNAs expression caused by alcohol. The mechanism of miRNAs alteration has not yet been fully understood. It is believed that the reduction in methylation levels of DNA is related to such alteration (26). We did not find in the literature studies that relate the expression of miRNAs in penile cavernous tissue associated with alcoholism and diabetes.

Yeligar et al. (27) performed a study using real-time PCR and western blotting in human endothelial cell culture and hepatic endothelial cells of rats. The control group was compared to the group submitted to alcoholism for 9 weeks, while human dermal microvasculature cells were exposed for 24 h to the effects of alcohol. They found an increase in ET-1 and its type B receptors as an effect of alcohol exposure, correlating the suppression of ET-1 expression by miR-199 in hepatic endothelial cells with miR-199 and miR-155 in human endothelial cells, thus indicating that these miRNAs may function as negative regulators of ET transcription control. Similarly, we found an increase in ET receptors in immunohistochemistry as an effect of alcohol, diabetes and their association. Similar results were found with decreased miR-199 expression in the corpus cavernosum of rats in the alcohol and alcohol + diabetes groups, which corroborate the hypothesis that increased ET could be determined by the suppressive action of miR-199 that is hypoexpressed due to the deleterious effects of alcohol.

Fichtlscherer et al. (28) studied plasma expression of some microRNAs known to be associated with endothelium and inflammation using quantitative PCR in patients with stable coronary disease comparing results with healthy volunteers and found significant hypoexpression of miR-199a and miR-155 in diabetics and males. Despite different species, our study also found an association of diabetes with the decrease in the expression of these microRNAs.

Our results showed that chronic ethanol consumption associated with diabetes changed the endothelinergic system in the CSM and played a role in the pathogenesis of ED. We can also suggest that changes in ET_A_ and ET_B_ receptors were regulated by miRNAs-155 and -199. The continued development of genomics offers new diagnostic methods and markers for complex diseases such as diabetes and alcoholism, as well as for their complications. Future *in vivo* studies can identify and test target molecular therapies for numerous disorders including ED as well as their possible associations with other conditions such as alcoholism and diabetes. Large-scale studies will also favor the identification of new miRNAs as targets for future studies.
